# Predictability of Neutrophil to Lymphocyte Ratio in preoperative elderly hip fracture patients for post-operative short-term complications: a retrospective study

**DOI:** 10.1186/s12891-023-06211-5

**Published:** 2023-03-25

**Authors:** Mohammad Golsorkhtabaramiri, John Mckenzie, Jan Potter

**Affiliations:** Aged Care Department, Illawarra and Shoalhaven Local Health District, Wollongong Hospital, Wollongong, New South Wales Australia

**Keywords:** Hip fracture surgery, Neutrophil to lymphocyte ratio, Elderly, Complication

## Abstract

**Purpose:**

Neutrophil to Lymphocyte Ratio (NLR) is a simple biomarker of systemic inflammatory response. We investigated predictability of NLR for early adverse outcome after surgery for hip fracture in elderly population.

**Methods:**

We reviewed a total of 971 elderly patients with hip fracture who underwent emergency surgery between January 2017 and July 2020 in the Department of Orthopaedics Surgery at the Wollongong Hospital. After considering exclusion criteria, data from a total of 834 patients included in our study. Socio-demographic data, NLR in admission, updated Charlson comorbidity index (uCCI), biochemical markers, mortality rate and 15 short term post-operative complications were collected to determine predictability of NLR for post-operative complications and mortality.

**Results:**

After hip surgery, Hospital in-patient case fatality rate was 3.7% (31). In addition, 63.1% (511) of the patients had at least one complication or more. Logistic regression demonstrated that raised NLR (*P*-value < 0.001, OR 1.05) and uCCI≥4 (*P*-Value < 0.001, OR 1.75) are associated with post-operative complications. Moreover, decreased haemoglobin was associated with adverse effects (*P*-value < 0.001, OR 0.97). No association was found for any of these variables with in-patient mortality except for albumin (*P*-value: 0.03). In addition, despite significant association, ROC analyses showed a low predictability for each of the above variables including NLR (AUC 0.59) for post-operative complications.

**Conclusions:**

Despite significant association, NLR was unable to prognosticate early adverse outcomes. However, it can be considered as a risk factor in admission for postoperative complications in combination with other risk factors and clinical context.

## Background

There are about 28,007 hip fractures a year in people over 65 years old in Australia [1]. This costs over one billion dollars a year for the Australian health care system which is almost 44% of fracture-related expenditure [2]. Higher comorbidities in elderly patients significantly increase the chance of post-operative complications and mortality [3]. Screening at-risk patients is vital for the health care team and will help in discharge planning and predicting outcome [4]. The main challenge faced by many clinicians is to identify the elderly patient with the lowest functional reserve. There are several scoring systems available for predicting 30-day and 1-year mortality risk including the Nottingham hip fracture score [4–6]. However, prognostication is always difficult and complicated even after using these scoring systems [3, 7]. Using inexpensive laboratory biomarkers for risk stratification before hip fracture surgery has recently been a topic of interest [8, 9]. Neutrophil to Lymphocyte Ratio (NLR) is a simple biologic indicator for systemic inflammatory response. In recent years, there has been an increasing interest in using NLR in the prognostication of cancer survival [10–14]. There is a growing body of literature that recognizes the importance of NLR in the prediction of complications after cardiac and vascular surgery [15–17]. However, most studies in this field have only focused on elective non-orthopaedic surgeries in a younger patient population. To date, there is little published data that have investigated the association between NLR and adverse outcomes in emergency hip fractures [18]. It is still not known whether NLR in admission can be considered a risk factor for postoperative complications in hip fracture of the elderly population. Our main aim in this project is to ascertain whether NLR can be used as a predictor for short term adverse outcomes in elderly patients after hip fracture surgery.

## Methods

This study is following the STROBE guideline [19].

### Study design

Retrospective observational single centre study.

### Setting

We included all emergency patients with hip fractures who underwent surgery between January 2017 and July 2020 at Wollongong Hospital orthopaedic department. Patients’ information was extracted manually by the Author from electronic Medical Records (eMR). Anonymous patient data was used retrospectively and no blood test was performed on any patient for this study. Follow up finished at the date of patient discharge to peripheral hospital from acute ward or death.

### Participants

A total of 971 consecutive patients who required emergency surgery after hip fracture with age of 65 years old or older were included in the study. We excluded patients with pathological fractures, active cancer or steroid treatment because of the potential bias of diluting results due to the established NLR prognostication in oncologic mortality in previous research [10–14]. Of note, distal femur fractures were excluded.

The surgical protocol consisted of early discharge from the Emergency department and surgery within 48 hours of arrival to the hospital unless there was an acute medical reason. After the operation, early mobilization and optimal pain control were performed with help of a multidisciplinary team of Geriatricians, Orthopaedic surgeons, acute pain team, physiotherapists and occupational therapists familiar with orthogeriatric medicine.

### Variables

The first Peripheral venous blood EDTA based sample was collected from each patient upon admission to the emergency department before surgery. These samples were analysed using a blood analyser in our laboratory for electrolytes, renal (creatinine, urea) markers and albumin (DxH 900 Haematology Analyzer, Beckman-Coulter, Inc., Brea, CA). Full blood count was analysed using standard automated laboratory methods (Cobas® 6000 Analyzer series, Roche Diagnostics, Tokyo, Japan) for haemoglobin, neutrophil and leucocytes counts. Age, gender, type of surgery and postoperative complications were collected manually from patients’ records in eMR. The NLR ratio was calculated by dividing absolute neutrophil counts by lymphocyte counts. In order to capture a wide variety of adverse outcomes, fifteen different types of complications were identified including (1) Severe arterial hypertension requiring medical intervention, (2) Decompensated heart failure requiring medical intervention, (3) Pulmonary oedema requiring medical intervention, (4) ST Elevation Myocardial Infarction(STEMI) and Non-STEMI, (5) Arrhythmia, (6) Thrombo-embolic complications, (7) Infections documented by radiological, biochemical and/or microbiological evidence, (8) Acute respiratory distress (other than 2,3 and 7), (9) Neurological dysfunction (stroke, alcohol withdrawal syndrome and epilepsy), (10) Electrolytes disturbance requiring treatment, (11) Anaemia- New onset requiring red blood cell transfusion, (12) Acute renal failure (evidenced by a new decline in eGFR), (13) Acute liver failure, (14) Direct Surgical complication and (15) Non- specified other complications which are not included in above categories. The rate of in-hospital death was calculated for the duration of the total length of orthopaedic admission to qualify for short term mortality.

Updated Charlson comorbidity index (uCCI) [20–22] was calculated manually based on preoperative comorbidities based on patient records. The calculation was based on 37 points scale with one point each for acute myocardial infarction, congestive heart failure, cerebrovascular accident, peripheral vascular disease, chronic obstructive pulmonary disease, connective tissue disease, dementia, peptic ulcer disease, Point 0 to 3 for mild to severe liver disease, 0 to 2 for diabetes mellitus, Point 0 to 6 for none, localised and metastatic solid tumour, 2 points for leukaemia and lymphoma and 6 points for diagnosis of AIDs.

### Data sources

The names of the patient was collected from local hip fracture registry. The data was collected from electronic Medical Records (eMR) which is a state-wide digital patient medical record used in public hospitals in New South Wales, Australia. Demographic data, date and type of surgery, post-operative complications, albumin level, haemoglobin level, NLR Day 0 (NLR0), mortality and complications during hospital stay were collected manually by the Author from eMR.

After collecting information, the population was divided into two groups of survived and non-survived according to in-patient mortality. Also, for sake of complications, the survived group was separated into two groups with and without complication.

### Statistical method

Descriptive analysis was used for quantitative variables such as age, gender and NLR0. Standard deviation and means have been used to describe continuous variables and for categorical variables, frequencies and percentages reported. We compared continuous variables with Students independent t-test and categorical variables with the Chi-square test. Test of equal variances was carried out before t-test for continuous variables. When there was a violation of the assumption of equal variances, we used non-parametric tests (Mann-Whitney). Following testing for the presence of differences, we used multiple logistic regression analysis to investigate the association of NLR with post-operative complications and mortality after adjustment for other variables. We used the updated Charlson index in our model instead of the ASA (American Society of Anaesthesiology) score because it would have increased the chance of collinearity. Updated CCI numbers equal to or more than 4 were considered high in the logistic regression model, based on previous literature [23]. The Performance of each variable in the prediction of postoperative short term complication and mortality is determined by calculating the area under the curve (AUC) using Receiver Operating Characteristics (ROC) analysis. Previous studies proposed NLR > 5 as a significant predictor for the adverse outcome [18, 24]. Results were considered statistically significant if 2-tailed *P*-value found to be under 0.05. Data analysis were performed using Jamovi 1.6 (Sydney, Australia) package [25] and SPSS version 27.0 software (SPSS Inc., Chicago, IL, USA). The statistical analysis was done by assistance of research clinicians in The Illawarra Health and Medical Research Institute (IHMRI).

## Results

### Participants

**Fig. 1:**
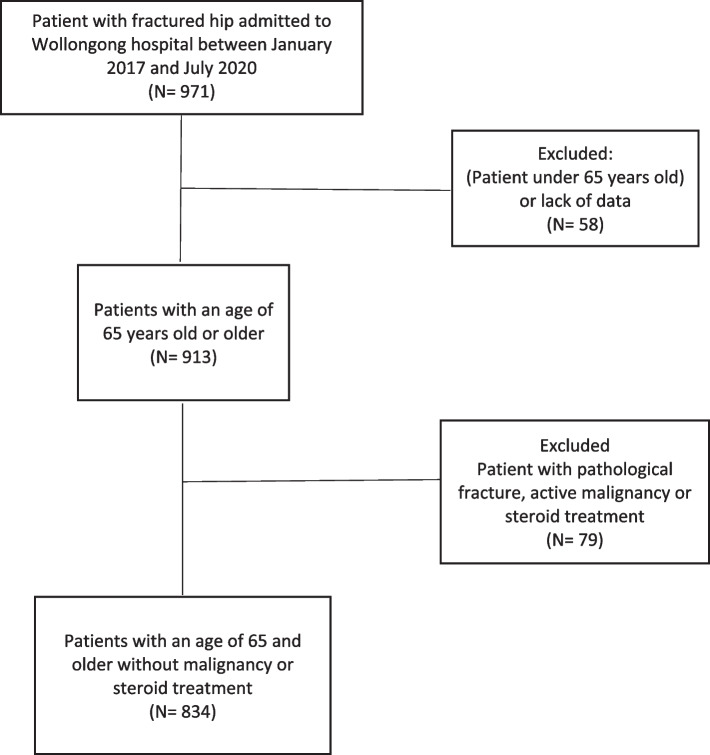
Flowchart for exclusion criteria

From a total of 971 patients with hip fractures who were admitted during our study timeline, 913 were 65 years old or older. In addition, 79 patients were excluded due to the presence of active malignancy, or steroid treatment. A total of 834 patients were eligible and included in our study (Flow chart in Fig. 1).

### Descriptive data

There were 597(71.6%) female and 237(28.4%) male patients. The patients’ average age was 84.1 ± 7.55 (range 65–107). A total of 511 (61.3%) patients had at least one complication and more. There was 31 (3.7%) death in hospital postoperatively. The average mean age in surviving patients was 84 ± 7.57 (Range 65–107) versus 86 ± 6.71 in non-survivors. The most common type of hip fractures were intertrochanteric (54.9%) followed by undisplaced intracapsular (23%), displaced intracapsular 16.1% and subtrochanteric (6%). Long Intramedullary nail was the most frequent surgery used (21% long nail vs 6.5% short nail). The next was hemiarthroplasty (19.2% cemented vs 12.8% uncemented) followed by sliding hip screw (12.9%) and total hip replacement (2.1% cemented vs 4.3% uncemented).

### Main results

Table 1 provides a comparison between complicated and non-complicated groups. What stands out in the table is that NLR0 was significantly higher in the complicated group (8.99 ± 5.73 vs 7.54 ± 5.63, *P*-value < 0.01). Also, albumin and haemoglobin were significantly lower in patients with at least one complication. (*P*-value 0.012 and 0.001 respectively).

**Table 1:** Descriptive results of hip fracture based on complications

Variables	Without complication	With one complication or more	*P* value
	*Mean ± SD or %*	*Mean ± SD or %*	
**Age**	83.3 ± 8.03	84.6 ± 7.18	0.04
**Gender (Female)**	228(27.3%)	369(44.2%)	0.613
**NLR 0**	7.54 ± 5.63	8.99 ± 5.73	0.001
**Albumin**	34.4 ± 5.63	33.3 ± 5.94	0.012
**Haemoglobin**	127 ± 15	120 ± 17.9	0.001

*P* <  0.05 is considered statistically significant

*Abbreviations*: *NLR* Neutrophil to lymphocyte ratio Day 0, *SD* standard deviation

In contrast, as shown in Table 2, for non-survivor and survivor groups, t-tests didn’t find any significant differences in mean scores of NLR0 for in-patient mortality. Moreover, no significant difference was detected for age or haemoglobin between the two groups. The only exception was albumin which was significantly lower in patients who died in the hospital (33.82 ± 5.85 vs 30.7 ± 4.8, *P*-value < 0.004).

**Table 2:** Descriptive results of Hip fracture based on mortality

Variables	Survived	Died	*P* value
	*Mean ± SD or %*	*Mean ± SD or %*	
**Age**	84.05 ± 7.57	86 ± 6.71	0.165
**Gender (Female)**	579 (97%)	18 (3%)	0.08
**NLR 0**	8.39 ± 5.78	9.54 ± 4.13	0.274
**Albumin**	33.82 ± 5.85	30.7 ± 4.8	0.004
**Haemoglobin**	123.14 ± 17.01	117.03 ± 19.96	0.052

*P* <  0.05 is considered statistically significant

*Abbreviations*: *NLR* Neutrophil to lymphocyte ratio Day 0, *SD* standard deviation

Moreover, Mortality or postoperative complications were not significantly different between male and female patients (*P*-value: 0.08 and 0.613 respectively).

Finally, we combined NLR0, age, sex, albumin, uCCI and haemoglobin in a logistic regression model to determine association with early postoperative complications. The results of the model as demonstrated in Table 3, showed raised NLR (OR 1.05, [95% CI 1.02–1.08], *P*-value < 0.001) and uCCI≥4 (OR 1.75, [95% CI 1.2–2.4], *P*-Value < 0.001) are independently associated with postoperative complications. Moreover, lower haemoglobin was associated with adverse effects (OR 0.97, [95% CI 0.97–0.98], *P*-value < 0.001).

**Table 3:** Multiple logistic regression for post-operative complications

Variables	OR	95% CI	*P* value
**Age**	1.004	0.97–1.02	0.71
**Gender (Female)**	0.91	0.65–1.2	0.72
**NLR 0**	1.05	1.02–1.08	< 0.001
**Albumin**	0.98	0.96–1.01	0.32
**Haemoglobin**	0.97	0.97–0.98	< 0.001
**uCCI ≥4**	1.75	1.2–2.4	< 0.001

*P* <  0.05 is considered statistically significant

*Abbreviations*: *uCCI* updated Charlson comorbidity index, *NLR 0* Neutrophil to lymphocyte ratio Day 0, *OR* odds ratio, *CI* confidence interval

We also ran our logistic model to determine the association of the above variable with in-hospital mortality. In contrast, this time, our model did not show any association of these variables with in-patient mortality except for albumin (OR 1.07, [95% CI 1.07–1.16], *P*-value: 0.03) (Table 4).

**Table 4:** Multiple logistic regression for in-hospital mortality

Variables	OR	95% CI	*P* value
**Age**	0.99	0.93–1.05	0.99
**Gender (Female)**	0.53	0.58–0.27	0.16
**NLR 0**	0.96	0.91–1.02	0.25
**Albumin**	1.07	1.07–1.16	0.03
**Haemoglobin**	1.01	0.98–1.03	0.23
**uCCI ≥4**	1.55	0.58–4.13	0.37

*P* <  0.05 is considered statistically significant

*Abbreviations*: *uCCI* Updated Charlson comorbidity index, *NLR 0* Neutrophil to lymphocyte ratio Day 0, *OR* odds ratio, *CI* confidence interval

**Fig. 2:**
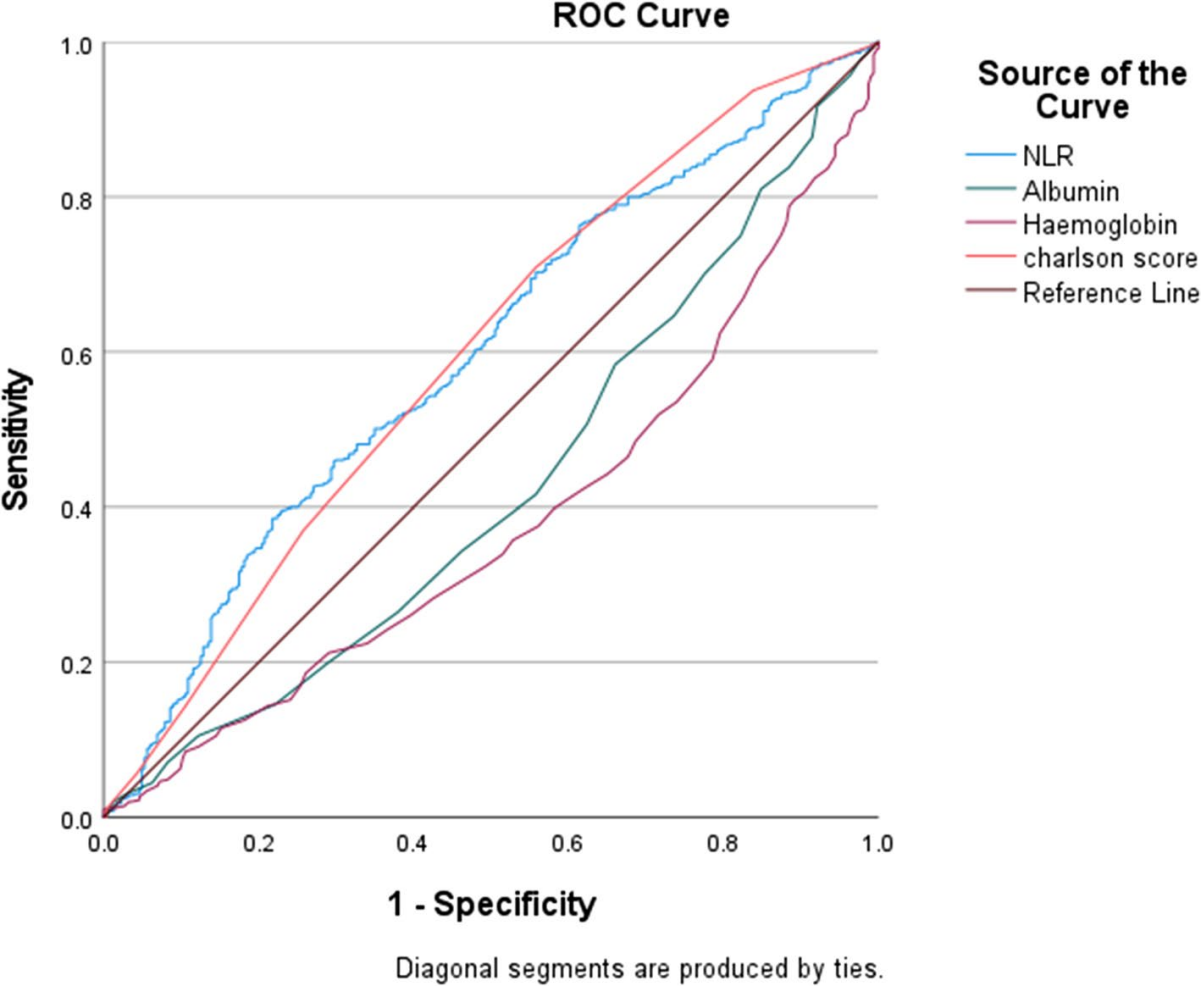
ROC curve of NLR, Albumin, Haemoglobin and uCCI. *Abbreviations*: ROC, receiver operator curve, uCCI, updated Charlson comorbidity index

Furthermore, to show prognostication, ROC analyses were performed which showed a low performance for each of the above variables in predicting postoperative complications (Fig. 2 and Table 5).

**Table 5:** Area under the curve, 95% confidence interval of NLR, Albumin and Charlson comorbidity index

Variables	AUC	95% CI	*P* value
**NLR 0**	0.59	0.55–0.63	< 0.00
**Albumin**	0.42	0.38–0.46	< 0.001
**Haemoglobin**	0.38	0.34–0.46	< 0.00
**uCCI**	0.59	0.55–0.63	< 0.00

*P* < 0.05 is considered statistically significant

*Abbreviations*: *uCCI* Updated Charlson comorbidity index, *NLR 0* Neutrophil to lymphocyte ratio Day 0, *OR* odds ratio, *CI* confidence interval, *AUC* area under the curve

## Discussion

### Main finding

As mentioned in the literature review, a few studies have shown an association of NLR with postoperative complications after hip fracture surgery [18, 24, 26]. The purpose of the current study was to determine the ability of admission NLR in predicting adverse effects after hip fracture in elderly patients. The most obvious finding to emerge from the analysis is that higher NLR in admission is significantly associated with postoperative complications. However, despite significant association, NLR0 has poor performance in predicting adverse outcomes (AUC 0.59). Taken together, these results suggest that NLR is an independent risk factor rather than a prognostic factor to predict outcome in a preoperative setting.

### NLR, risk factor and prognosis

Considering NLR as an independent risk factor, our finding broadly supports the work of other research in this area [18, 24]. Forget et al. [24, 26] previously studied 247 elderly patients after hip fracture surgery. The authors concluded that NLR is an independent risk factor for postoperative complications (OR: 3.34, [95% CI 2.33–4.80], *P*-Value 0.001). However, in their multivariate analysis, they considered NLR at day five as a significant risk factor that has a lower clinical value than NLR in admission. In our study, we demonstrated that NLR in admission can also be considered a risk factor even in the preoperative period in correlation with clinical assessment.

Although extensive research has been carried out on the prognostication of NLR in the field of cardiac or vascular surgery, a small number of studies exists in the field of hip fracture surgery. Previously, Fisher and colleagues [24] reported NLR in admission as a modest predictor of postoperative complications. This is contrary to our finding which found it as a poor predictor. A possible explanation for this might be that they considered mortality, postoperative myocardial injury (raised Troponin), high inflammatory response (increased CRP) and length of stay> 10 days as adverse outcomes. Our definition of short-term outcomes was different from their study. We measured a wide range of clinical and biochemical complications which occurred in a short period after hip fracture surgery.

### NLR and mortality

Mortality after hip fracture surgery varies from 3.4% [2] in Australia to 19.5% in other countries [27]. In our study in-patient mortality was 3.7% which is in line with the general pattern in Australian Hospitals. We were unable to demonstrate any prediction capacity of NLR0 for in-patient mortality. This accords with earlier observations with those of previous studies [3, 18]. Niessen et al. reviewed 362 hip fracture patients and concluded NLR in admission doesn’t have any discriminative ability in predicting mortality after hip fracture surgery (CI 0.058 ± 0.038, *P*-value 0.12) [3]. However, this finding is contrary to Fisher et al. [24] which showed a higher chance of mortality in patients with NLR > 8.5 compared to patients with NLR of 5.1–8.5. This inconsistency may be explained by their mixed case selection strategy. They included all the patients with elective surgery, suspected surgical site and prosthetic joint infections in their study. It is different from our cohort and that of Niessen et al., which focused only on the emergency hip fractures.

However, This discrepancy between studies shows that these results need to be interpreted with caution and further studies need to be conducted to carefully examine mortality risk.

### Other variables

In our study, we showed that higher uCCI is a risk factor for postoperative complications. It is interesting to note that NLR0 and uCCI had the same predictability according to the ROC graph (AUC: 0.59) (Fig. 2). Surprisingly, contrary to our expectations, uCCI had a weak association with in-patient mortality despite previous studies [28]. This discrepancy could be attributed to the concept of frailty in elderly patients. None of these risk factors is measuring frailty which is the main contributing factor for functional reserve in hip fracture patients. This idea is supported by previous studies which showed a lack of performance in multiple geriatric scores in predicting postoperative outcomes [29].

### NLR pathophysiology

The possible mechanism of NLR association with adverse outcomes is not entirely clear. It is demonstrated that Neutrophil extracellular traps (NETs) which contain mitochondrial DNA (mt DNA) are present for 5 days after major orthopaedic surgery [30]. They represent a form of an independent inflammatory response as a marker of an augmented innate immune system. They are different from nuclear DNA which is released from NETs in sepsis [31]. Further in-vitro studies show that mtDNA can activate Stimulator interferon gens(STING), Toll like receptor 9 (TLR9), extracellular signal-regulated kinase 1/2 (ERK1/2) and p38 mitogen-activated kinase (MAPK) signalling pathways which consequently lead to ‘NETosis’ [32]. Previous studies showed the role of NETosis in the formation of deep venous thrombosis in trauma patients [33]. Therefore, these finding suggests a different inflammatory response leading to postoperative complications, independent from other risk factors in hip fracture patients.

In addition, lymphopenia has been an indicator of malnutrition in hip fracture patients [34]. Blanco et al., [35] recently found there is a significant increase in 30-day mortality in patients with lymphocytopenia (OR 1.842 [CI 1.063–3.191], *P*-value 0.029). These results corroborate a great deal of the previous work with d’Engremont et al. [36] and Imabayashi et al. [37] who referred to lymphopenia as a risk factor for postoperative mortality. It suggests that low lymphocyte count as a denominator in NLR can have some implications for malnutrition and mortality in hip fracture patients.

However, it is important to bear in mind that elevated NLR0 in the emergency hip fracture is considered an acute inflammatory response and can be affected by a variety of confounding factors.

### Limitations and strengths

The most important limitation for this study lies in the fact that firstly, this is a retrospective observational study with limited power for causality. Secondly, we didn’t include postoperative delirium in the list of complications due to retrospective measurement limitations. Some studies consider NLR as a predictor for delirium [38]. Thirdly, immune system impairment in the elderly population and multifactorial response to fracture can affect neutrophil and lymphocyte count. Fourthly, we didn’t exclude patients who were active smokers who could have higher neutrophil counts.

In terms of strengths, we used a large sample of patients to decrease the chance of selection bias. We also excluded patients with cancer and those on steroid treatment which could be a field for confounders due to the known effect of raised NLR in the oncologic population.

## Conclusion

We demonstrated that NLR can be considered as a risk factor in admission for postoperative complications in combination with other risk factors. However, it failed to predict short term in-patient adverse outcomes accurately as a sole predictor in admission. More research using controlled trials is needed to include serial NLR in perioperative patients as well as looking into other predictors for hip fracture surgery complications.

## Data Availability

The datasets generated and/or analyzed during the current study are not publicly available due lack of patient’s consent but are available from the corresponding author on reasonable request.
